# Successful optical genome mapping from 500 000 cells: A low-input UHMW DNA extraction approach

**DOI:** 10.1016/j.mex.2025.103742

**Published:** 2025-11-29

**Authors:** Elly De Vlieghere, Friedel Nollet, Helena Devos, Barbara Cauwelier

**Affiliations:** Department of Laboratory Medicine, AZ Sint-Jan Brugge, Bruges, Belgium

**Keywords:** Optical genome mapping, Ultra-high molecular weight DNA, Hematology, Cytogenetics, Low cell number, CD138 selected cells

## Abstract

Optical Genome Mapping (OGM) is an emerging technology in clinical laboratories for identifying copy number and structural variations in the DNA of patients with haematological malignancies. A critical initial step is the isolation of ultra-high molecular weight genomic DNA (UHMW gDNA), which typically requires 1.5 million white blood cells. However, this cell number is not always achievable in clinical practice due to various limitations. For instance, diagnostic analysis of multiple myeloma (MM) is should be performed on CD138-positive cells derived from bone marrow aspirates (BMA), where both the sample volume and the number of CD138-positive cells

This method describes a customized protocol which enables isolation of UHMW gDNA starting from as few as 500 000 cells, while still resulting in DNA of sufficient quality and quantity to perform OGM and collect at least 1500 Gbp of data.

## Graphical abstract

**Figure:**
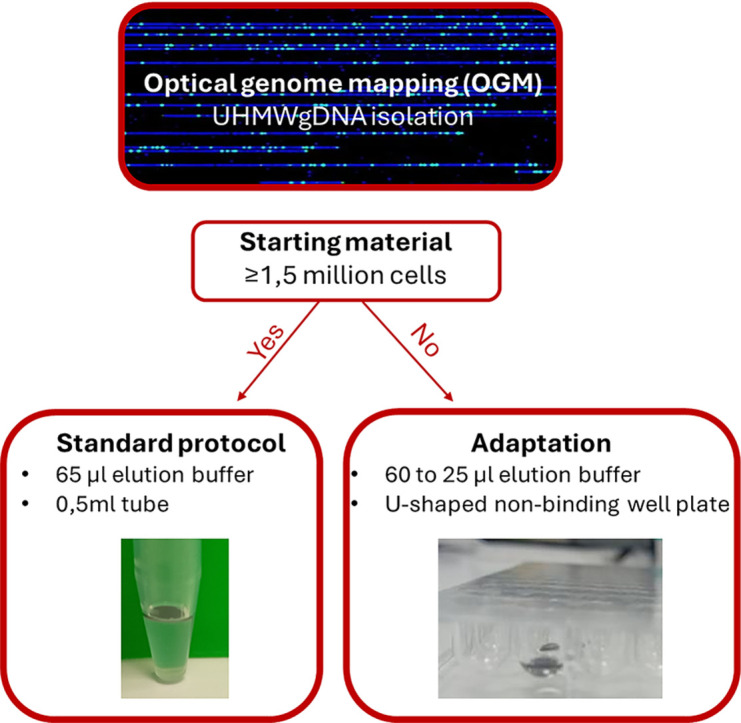

Key advantages:

• Optimal use of limited patient material
• Reliable isolation of high-quality and sufficient quantity UHMW gDNA for OGM
• Reduced input requirement from 1.5 million to 500 000 cells

**Specifications table**

**Table:** 

**Subject area**	Medicine and Dentistry
**More specific subject area**	Cytogenetics
**Name of your method**	Isolation of ultra-high molecular weight DNA for Optical Genome Mapping with minimal input material
**Name and reference of original method**	Isolation of ultra-high molecular weight DNA for Optical Genome Mapping.
	H. Yang, G. Garcia-Manero, K. Sasaki, G. Montalban-Bravo, Z. Tang, Y. Wei, T. Kadia, K. Chien, D. Rush, H. Nguyen, A. Kalia, M. Nimmakayalu, C. Bueso-Ramos, H. Kantarjian, L.J. Medeiros, R. Luthra, R. Kanagal-Shamanna, High-resolution structural variant profiling of myelodysplastic syndromes by optical genome mapping uncovers cryptic aberrations of prognostic and therapeutic significance, Leukemia 36 (2022) 2306–2316. https://doi.org/10.1038/s41375-022-01652-8.
	K. Rack, J. De Bie, G. Ameye, O. Gielen, S. Demeyer, J. Cools, K. De Keersmaecker, J.R. Vermeesch, J. Maertens, H. Segers, L. Michaux, B. Dewaele, Optimizing the diagnostic workflow for acute lymphoblastic leukemia by optical genome mapping, Am J Hematol 97 (2022) 548–561. https://doi.org/10.1002/ajh.26487.
**Resource availability**	This method is an adaptation of the manufacturers recommendations to isolate ultra-high molecular weight DNA from 1.5 million cells. Standard procedures can be found on the website of the manufacture (Bionano): https://bionano.com/sample-preparation-kit-support/

## Background

Optical Genome Mapping (OGM) is a high-resolution, genome-wide analysis technique used to detect copy number variations (CNVs) and structural variations (SVs) in DNA. These structural variations include large insertions, deletions, duplications, inversions, and translocations. The process begins with the isolation of ultra-high molecular weight genomic DNA (UHMW gDNA), typically consisting of fragments hundreds of kilobases in length. These DNA molecules are fluorescently labeled at specific sequence motifs, producing unique “barcode” patterns for each fragment. The labeled DNA is then linearized within nanochannels and visualized using a high-resolution optical imaging system. The resulting fluorescent barcodes are digitally assembled and aligned to a reference genome. Structural and copy number variants are identified by comparing the barcode patterns to that of the reference genome. OGM is considered complementary to next-generation sequencing (NGS), and together these techniques offer a near-complete view of the genome.

Hemato-oncology is one of the clinical fields in which OGM has demonstrated significant diagnostic utility. Diagnosis is typically based on bone marrow aspirates (BMA) or lymph node biopsies. In multiple myeloma (MM) and in lymph node biopsies, diagnostic material is often limited, especially in MM samples where cytogenetic analysis is based on CD138-positive plasma cells isolated from BMAs using magnetic bead separation. The standard OGM protocol requires a minimum of 1.5 million cells. In routine clinical practice, however, this quantity cannot always be attained.

Applying the standard protocol to lower cell inputs frequently results in UHMW gDNA of suboptimal concentration and/or fragment length. To overcome this limitation, we optimized the existing protocol to enable high-quality UHMW gDNA extraction from as few as 500 000 cells, while still achieving sufficient DNA quality and quantity for reliable OGM analysis.

## Method details

**Fig. 1:**
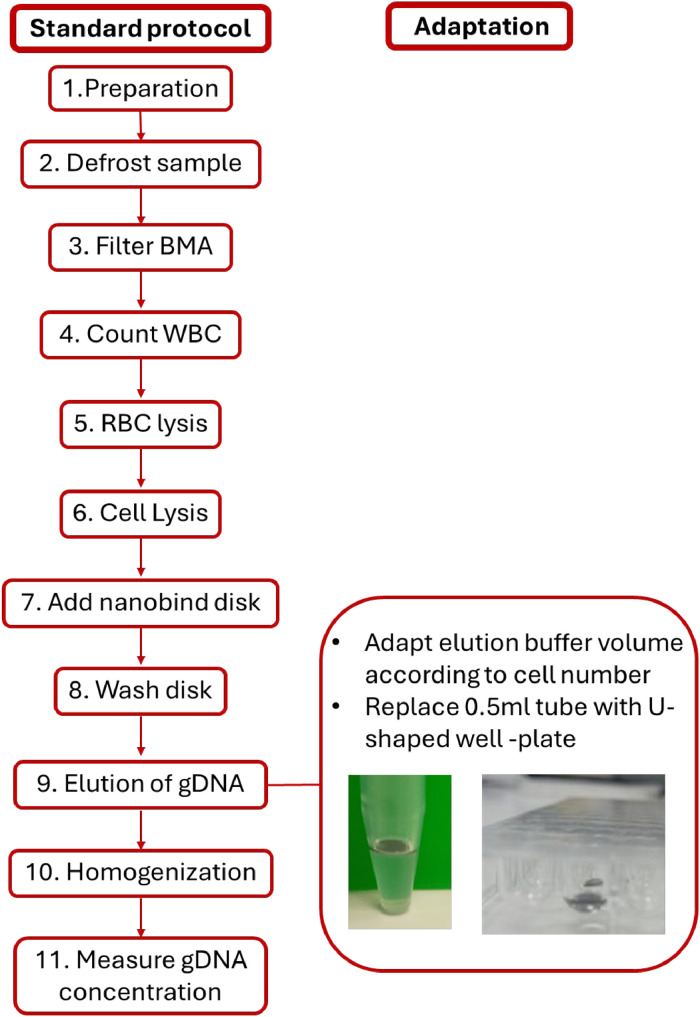
Schematic overview of the DNA isolation protocol, highlighting the adaptation made during the elution step.

The procedures are performed according to the manufacturer's protocol (Bionano) [[1], [2]]. However, Step 9-Elution of gDNA- was customized to enable the isolation of high-quality UHMW gDNA from a limited number of cells (Fig. 1). When using the standard protocol with low-input samples, the resulting gDNA concentration is often insufficient for successful OGM analysis.

Our modification to the elution step (highlighted in bold) ensures both a sufficient gDNA concentration and high molecular weight integrity. This adaptation enables reliable and high-quality OGM even when starting with a limited cell count.

### Sample storage

The isolation procedure can be initiated from either fresh or frozen samples. The method has been validated using frozen samples stored at −80 °C, following the manufacturer's recommendations (Bionano). Storage procedures are described below for different sample types:

• Bone Marrow Aspirate (BMA) Collected in EDTA Tubes
  ○ Mix the sample on rollers for 15 min at room temperature.
  ○ Transfer 1 mL of BMA to a freezing tube and add 15 µL of DNA Stabilizer (Bionano, 20398).
  ○ Store at −80 °C.
• Single-Cell Suspension from Tissue (e.g., Biopsy) or CD138⁺-Selected Cells
  ○ Centrifuge cells for 5 min at 375 × *g* at 4 °C.
  ○ Carefully remove the supernatant.
  ○ Resuspend the cell pellet in 1 mL of a 10 % DMSO in Alburex® 5 (CSL Behring) solution.
  ○ Transfer the suspension to a freezing tube.
  ○ Place the tube into a CoolCell® freezing container (Corning, 432004) and store at −80 °C. Follow the manufacturer's instructions to ensure controlled freezing.

### Isolation

DNA isolation is performed using the Bionano Prep SP-G2 Blood and Cell DNA Isolation Kit (Bionano, 80118) in combination with the Bionano Prep SP-G2 BMA Add-On (Bionano, 80062).

Protocol Steps

1. Preparation

• Cold Stabilizing Buffer (CSB):
  Prepare fresh and keep on ice. For 1 mL of CSB, mix:
  ○ 20 µL DNA Stabilizer (Bionano, 20469)
  ○ 980 µL Cell Buffer (Bionano, 20372)
• Lysis & Digestion Cocktail:
  For 1 sample, prepare just before use:
  ○ 270 µL Digestion Enhancer (Bionano, 20457)
  ○ 66.25 µL Ultrapure Water (Bionano, 20532)
  ○ Appropriate volumes of:
    ■ Lysis and Binding Buffer (Bionano, 20372)
    ■ DE Detergent (Bionano, 20461)
    ■ Thermolabile Proteinase K (Bionano, 20456) – *add immediately before use*

2. Sample Defrosting

• Bone Marrow Aspirate (BMA):
  ○ Defrost by incubating the sample at 37 °C for 2 min.
  ○ Immediately transfer to an ice bath.
  ○ Invert the tube 10 times and perform a quick pulse spin.
• Cell Suspension (e.g., CD138⁺ or biopsy-derived):
  ○ Defrost by incubating at 37 °C.
  ○ Monitor closely; once thawed, centrifuge immediately at 300 × g for 10 min at room temperature (RT).
  ○ Carefully remove the supernatant and resuspend the pellet in 1 mL of cold CSB.

3. Filtering BMA Samples

(Only applicable for BMA samples)

• Pipette a maximum of 500 µL onto a BMA Filter (Bionano, 20464) placed on a 2 mL collection tube (Bionano, 20452).
• Centrifuge at 400 × g for 5 min at RT.
• Resuspend the filtered cells in cell buffer.

4. White Blood Cell (WBC) Count

• Determine the WBC concentration using the HemoCue WBC system.
• If the concentration exceeds 30 × 10⁶ cells/mL, dilute the sample with Cell Buffer (Bionano, 20372) accordingly.

5. Cell Transfer and Pretreatment

• Transfer 1.5 × 10⁶ cells to a pre-cooled 1.5 mL Protein LoBind tube (Bionano, 20540).
  **If the total number of cells is** <**1.5 × 10⁶, transfer all available cells**.
• BMA samples only:
  ○ Add RBC Lysis Buffer (Bionano, 20442), using 3 × the sample volume.
  ○ Invert 10 ×, incubate at room temperature (RT) for 5 min, then invert another 10 × .
• Centrifuge at 2200 × *g* for 2 min at RT.
• Carefully remove the supernatant, leaving 40 µL in the tube.

6. Cell Lysis

• Add 20 µL of cold CSB. Mix gently by pipetting 5 times in a slow, rotating motion.
• Add 430 µL of Lysis & Digestion Cocktail. Invert the tube 15 times. Incubate on the HulaMixer (ThermoFisher, 15920D) at 10 rpm, 15 min, RT.
• Pulse spin briefly and incubate at 55 °C for 10 min.
• Only for magnetic bead–selected cells:
  ○ Remove magnetic beads using the DynaMag (ThermoFisher, 1231D) for 10 min.
  ○ The beads will adhere to the magnet. Carefully transfer the supernatant (containing the cells) to a new 1.5 mL LoBind tube (Bionano, 20540).

7. gDNA Binding to the Nanobind Disk

• Add a Nanobind Disk (Bionano, 20539) to the lysed sample.
• Add 480 µL of 100 % isopropanol, then invert the tube 5 times.
• Place on the HulaMixer at 10 rpm, 10 min, RT.

8. Washing the Nanobind Disk

  • Use the DynaMag (ThermoFisher, 1231D) to remove all liquid.
  • Add 700 µL Wash Buffer 1 (Bionano, 20458). Mix on HulaMixer at 10 rpm, 1 min, RT.
  • Repeat the previous steps one more time.
  • After final removal of Wash Buffer 1, add 500 µL Wash Buffer 2 (Bionano, 20460).
  • Mix on HulaMixer at 10 rpm, 1 min, RT and remove liquid using the DynaMag (Thermofisher, 1231D).

9. Elution of gDNA (Adapted Step)

• **When the WBC count in step 5a was > 1.2 × 10⁶, transfer the Nanobind Disk with a magnetic retriever to a 0.5 mL Protein LoBind tube (Bionano, 20541). If the WBC count in step 5a was < 1.2 × 10⁶, transfer the disk to a U-shaped non-binding well plate (Greiner Bio-One, 650901) instead.**
• **Spin down and remove all residual liquid.**
• **Add Elution Buffer (Bionano, 20463), based on the number of cells transferred in step 5a** (Fig. 2).

**Fig. 2:**
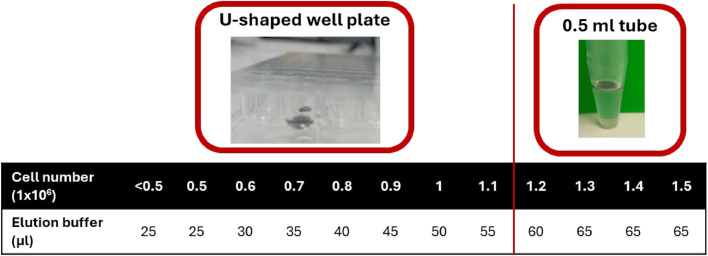
Adaptation of step 9: Elution of gDNA. Table with volume of elution buffer corresponding to the number of input cells.
• **Ensure the Nanobind Disk is positioned horizontally and fully immersed in the buffer.**
• Incubate for 20 min at room temperature (RT) to allow DNA elution from the Nanobind disk.
• Carefully transfer the viscous eluate to a 2 mL Protein LoBind tube (Bionano, 20542).
• Perform a short spin of the original tube or **plate** to collect any remaining liquid.
• Transfer this remaining volume to the same 2 mL tube.
• Repeat if necessary to ensure maximum recovery of gDNA**.**

10. Homogenization by Controlled Shearing

• **Set your pipette to the same volume as the elution buffer used in Step 9.**
• Gently pipette the gDNA up and down six times in a slow, circular motion, then spin down briefly.
• Incubate the samples overnight or over the weekend at room temperature (RT) in a light-protected box.

11. Measurement of gDNA Concentration

• Measure the gDNA concentration using the Qubit™ dsDNA Broad Range Assay Kit (ThermoFisher, Q32850).
• Perform measurements in triplicate. **If the elution buffer volume used is** <**55 µL, measure in duplicate.**
• The expected gDNA concentration should be between 36 and 250 ng/µL, with a coefficient of variation (CV) < 0.3.

## Method validation

### Sample information

**Fig. 3:**
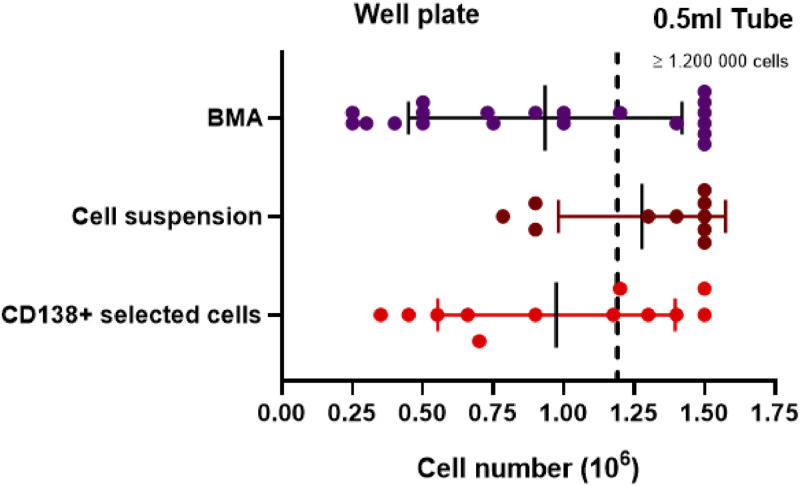
Distribution of the samples by cell number for each sample type. Samples with <1.2 million cells (dashed line) are eluted in a U-shaped well plate, samples with ≥ 1.2 million cells are eluted in a 0.5 ml tube.

A total of 42 samples were analyzed, comprising 20 bone marrow aspirates (BMA), 12 CD138+ magnetic bead-selected cells, and 10 cell suspensions obtained from lymph nodes, tissue biopsies, or pleural effusion samples (Fig. 3). The volume of elution buffer and the use of the U-shaped well plate are determined by the available cell number (Table 1). The optimal ratio between cell number and elution buffer volume, for samples with low cell counts was determined beforehand by trial and error on different samples. If the elution buffer volume is lower than 60 µl, the Nanobind Disk is not fully immersed when using an 0.5 ml tube. For this reason, all samples containing fewer than 1.2 million cells are eluted in a U-shaped well plate (Fig. 2). Our validation set contains 29 samples with fewer than 1.5 million cells, of which 22 were eluted in a U-shaped well plate (fewer than 1.2 million cells; Table S1 and Fig. 3).

### Molecule quality report (MQR)

The MQR is generated by the Bionano Access software and includes various quality metrics collected during an OGM run. These parameters allow assessment of the run quality, with recommended cut-offs provided by the manufacturer. We evaluated the effective coverage, DNA concentration and the fragment length to assess the quality of the isolated DNA.

**Fig. 4:**
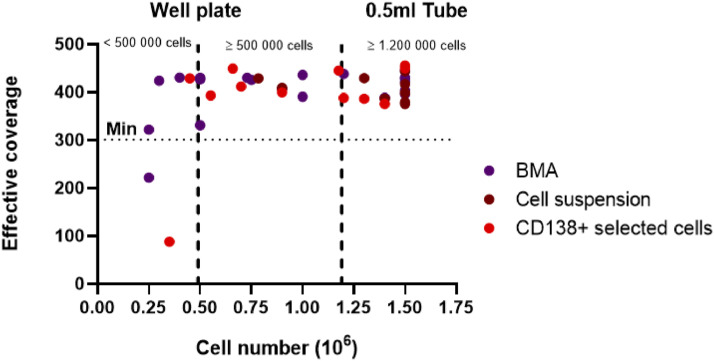
Effective coverage of the samples by cell number, colors indicate the sample type. The minimal requirement for effective coverage is 300X (dotted line). Samples with <1.2 million cells (dashed line) are eluted in a U-shaped well plate, samples with ≥ 1.2 million cells are eluted in a 0.5 ml tube.

The *Effective coverage* is the most comprehensive parameter for assessing OGM data quality. It represents the total size of all aligned DNA fragments divided by the size of the reference genome. An minimum post-analytical effective coverage of 300X is advised by the manufactures to ensure high-quality data analysis. This threshold is only achieved when both the DNA concentration is sufficient and the DNA fragments are of adequate length as only DNA fragments of at least 150 kbp are included in the mapping process. Among the samples eluted in the 0.5 mL tube (i.e. ≥1.2 × 10^6^ cells), all 20/20 (100 %) reached the required 300X effective coverage. Of the samples eluted using the U-shaped well plate (i.e. <1.2 × 10^6^), 20/22 (90 %) reached this threshold. When stratifying by cell input, 16/16 (100 %) of the well-plate eluted samples with ≥500 000 cells were successful, while only 4/6 (66 %) of those with <500 000 cells achieved the desired 300X effective coverage (Table S1 and Fig. 4).

### DNA concentration

**Fig. 5:**
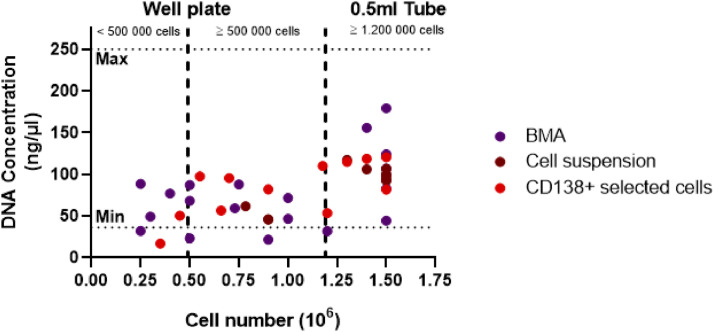
DNA concentration of the samples by cell number colors indicate the sample type. DNA concentration should be between 36 and 250 ng/µl (dotted line). Samples with <1.2 million cells (dashed line) are eluted in a U-shaped well plate, samples with ≥ 1.2 million cells are eluted in a 0.5 ml tube.

At the end of the DNA isolation protocol, gDNA concentration is measured. The recommended concentration, to starting with the labeling procedure is between 36 and 250 ng/µl. Among the samples eluted in the 0.5 ml tube, 19 out of 20 (95 %) had DNA concentrations within this recommended range. For the samples with cell amount between 1.2 × 10^6^ and ≥ 500 000, 14 out of 16 (87 %) met this criterion. In contrast, among the samples with < 500 000 cells, only 4 out of 6 (66 %) had DNA concentrations within the optimal range.

(Table S1 and Fig. 5).

### Length of DNA fragments

**Fig. 6:**
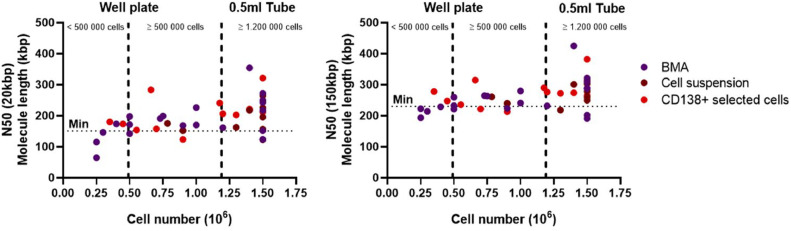
N50 (20 kbp) and N50(150 kbp) of the samples by cell number, colors indicate the sample type. DNA or molecule length should be at least 150 and 230 kbp for the N50 (20 kbp) and N50(150 kbp) respectively (dotted line). Samples with <1.2 million cells (dashed line) are eluted in a U-shaped well plate, samples with ≥ 1.2 million cells are eluted in a 0.5 ml tube.

Two MQR parameters describe the length distribution of isolated DNA fragments. N50 (20 kbp) and N50(150 kbp) which represent the average fragment length of all molecules with a minimal size of 20 kbp and 150 kbp respectively. These parameters are expected to be at least 150 kbp and 230 kbp respectively. For samples eluted in 0.5 ml tube samples (i.e. ≥1.2 × 10^6^ cells), 16 out of 20 (80 %) met both criteria. In contrast, among U-shaped well-plate eluted samples containing ≥ 500 000 cells, 10 out of 16 (62.5 %) succeeded, whereas only 2 out of 6 (33 %) samples with fewer than 500 000 cells met the thresholds (Table S1 and Fig. 6).

### Cell number series

BMA of a 29-year-old female ALL patient with 89 % blasts was used to perform a cell number series. UHMW gDNA isolation was performed as described above, starting from 1.5, 1, 0.75, and 0.5 million cells. Samples were diluted with cell buffer prior to isolation. This implies that the malignant cells/DNA were not diluted with normal cells/DNA.

**Fig. 7:**
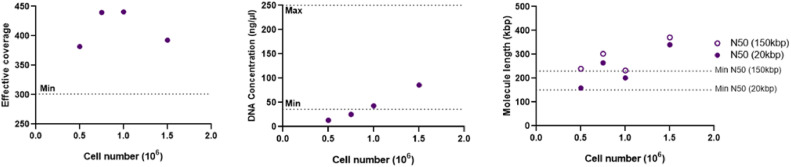
Effective coverage, DNA concentration, and molecule length of one patient sample starting isolation from 1.5, 1, 0.75, or 0.5 million cells. The dotted line indicates minimal and maximum advised values.

All four isolations easily reached the minimal effective coverage of 300x (Fig. 7); consequently, the runs were considered successful. Despite this, DNA isolation of the samples with 0.5 and 0.75 million cells failed to yield the desired 36 ng/µl DNA (Fig. 7). All four isolations reached the minimal molecule length; however, for the isolations with 0.5 and 1 million cells, the molecule lengths were borderline. Overall, these results are in line with the validation data above.

**Fig. 8:**
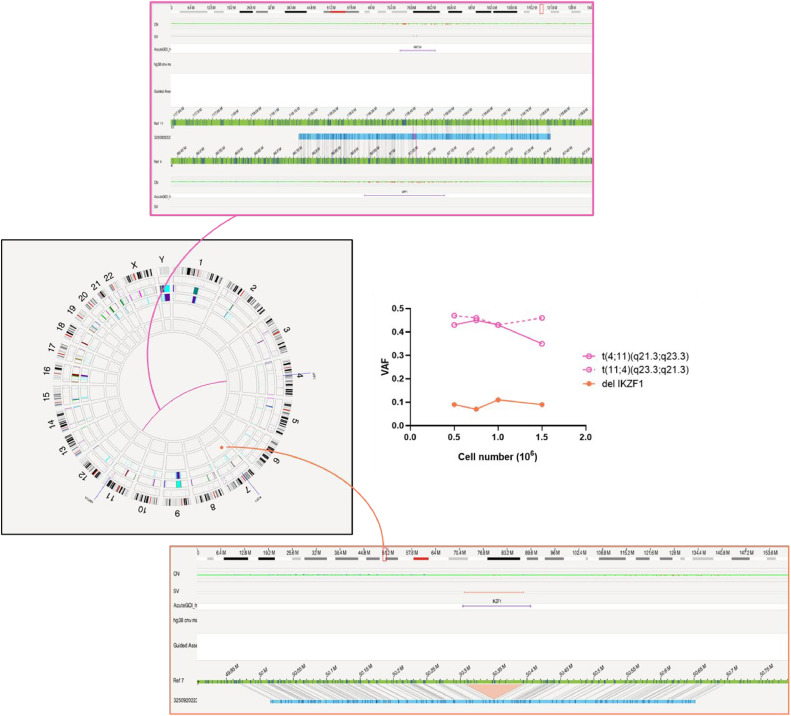
The detected variants t(4;11)(q21.3;q23.3) [*KMT2A:: AFF1*] (pink) and deletion *IKZF1* (orange) visualized in the Circos Plot (center) and Genome Browser (upper and lower panel). The graph in the centers contains the variant allele frequency (VAF) for the detected variants of one patient sample starting isolation from 1.5, 1, 0.75 or 0.5 million cells.

The raw data of these runs (molecule files) were analyzed with Bionano Access v1.8.2 using the Guided Assembly low allele frequency pipeline with hg38 as the reference genome. This patient has a deletion of *IKZF1* and a translocation t(4;11)(q21.3;q23.3), resulting in the fusion gene *KMT2A::AFF1*. All four isolations resulted in the detection of the same variants; only small variations between the exact breakpoints were observed. The variant allele frequency (VAF) of the *IKZF1* deletion varied between 0.07 and 0.11. The translocation t(4;11)(q21.3;q23.3) was detected along with its reciprocal t(11;4)(q23.3;q21.3), both were detected with VAFs between 0.35 and 0.47. In the isolation with 1.5 million cells, a VAF of 0.35 was detected for t(4;11)(q21.3;q23.3), while the reciprocal had a VAF of 0.46. This isolation had suboptimal quality labeling parameters what could explain the large variation. In the other isolations, the VAFs of both translocations were all between 0.43 and 0.47. Small variations in VAF are inevitable and not related to the cell number of the sample (Fig. 8).

### Summary and recommendations

This validation demonstrates that isolating UHMW gDNA for successful OGM from samples with only 500 000 cells, instead of the recommended 1.5 million cells for OGM, is feasible. The volume of the elution buffer is adapted to the available number of cells. Up to a volume of 60 µl, equivalent to an elution from 1.2 million cells, the 0.5 ml tube provided by the manufacturer can be used. Below this volume, we recommend eluting the gDNA from the Nanobind Disk in a U-shaped, non-binding well plate. In this plate, the Nanobind Disk remains fully immersed if at least 25 µl of elution buffer is used (Fig. 2).

With these adaptations, we observe a 100 % success rate (effective coverage of at least 300X) for samples containing between 500 000 and 1.2 million cells, despite DNA concentration and fragment length being slightly lower than in samples eluted in 0.5 ml tubes. When samples contain fewer than 500 000 cells, the elution buffer volume should be kept at 25 µl, as further lowering this volume cannot ensure that the Nanobind Disk remains fully immersed. Consequently, the success rate decreases for samples starting with fewer than 500 000 cells, although successful OGM still remains possible. Our adaptations do not dilute the samples with other cells or DNA; for this reason, there is no effect on variant detection.

We recommend following the manufacturer’s protocol and using 1.5 million cells whenever possible. However, when fewer cells are available, the adjustments described in our protocol allow for a significant reduction in the minimum required cell number to as low as 500 000 cells.

## Limitations

The cell numbers described above refer to counts obtained after thawing the samples. However, cell loss during freezing and thawing process must be considered. For single-cell suspensions we observe an average loss of approximately 30 %. Consequently, a minimum of 750 000 cells should be frozen to ensure successful isolation and OGM run. When sample cell numbers are very limited, it is preferable to start from fresh samples. However, isolation starting form fresh samples using the well plate method has not been validated within this study.

## Ethics statements

The study was conducted in compliance with the principles of the declaration of Helsinki, good clinical practice and all applicable regulatory requirements.

## Related research article

None

## For a published article

None

## CRediT author statement

Elly De Vlieghere: Conceptualization, methodology, validation, formal analysis, investigation, writing - original draft, visualization

Friedel Nollet: Conceptualization, writing - review & editing, funding acquisition

Helena Devos: Conceptualization, writing - review & editing, supervision, resources, funding acquisition

Barbara Cauwelier: Conceptualization, writing - review & editing, supervision, resources, funding acquisition

## Declaration of competing interest

The authors declare that they have no known competing financial interests or personal relationships that could have appeared to influence the work reported in this paper.

## Data Availability

Data will be made available on request.

## Appendix Supplementary materials

Image, image 1
